# Immunoregulatory mechanisms in Chagas disease: modulation of apoptosis in T-cell mediated immune responses

**DOI:** 10.1186/s12879-016-1523-1

**Published:** 2016-04-30

**Authors:** Ana Thereza Chaves, Juliana de Assis Silva Gomes Estanislau, Jacqueline Araújo Fiuza, Andréa Teixeira Carvalho, Karine Silvestre Ferreira, Rafaelle Christine Gomes Fares, Pedro Henrique Gazzinelli Guimarães, Elaine Maria de Souza Fagundes, Maria José Morato, Ricardo Toshio Fujiwara, Manoel Otávio da Costa Rocha, Rodrigo Correa-Oliveira

**Affiliations:** Laboratório de Imunologia Celular e Molecular, Centro de Pesquisas René Rachou, Fiocruz, Belo Horizonte, Brazil; Laboratório de Biologia das Interações Celulares, Departamento de Morfologia, Instituto de Ciências Biológicas, UFMG, Belo Horizonte, Brazil; Programa de Pós graduação em Medicina Tropical e Infectologia, Faculdade de Medicina, UFMG, Belo Horizonte, Brazil; Laboratório de Biomarcadores de Diagnóstico e Monitoração, Centro de Pesquisas René Rachou, Fiocruz, Belo Horizonte, Brazil; Laboratório de Imunologia e Genômica de Parasitos, Departamento de Parasitologia, Instituto de Ciências Biológicas, UFMG, Belo Horizonte, Brazil; Departamento de Fisiologia e Biofísica, Instituto de Ciências Biológicas, UFMG, Belo Horizonte, Brazil; Instituto Nacional de Ciência e Tecnologia em Doenças Tropicais – INCT-DT, Minas Gerais, Brazil; NUPEB, Universidade Federal de Ouro Preto, Ouro Preto, Brazil

**Keywords:** Chagas disease, Immunoregulation, Apoptosis, TNF/TNFR superfamily, Caspase family, T lymphocytes, *Trypanosoma cruzi*

## Abstract

**Background:**

Chronic Chagas disease presents different clinical manifestations ranging from asymptomatic (namely indeterminate) to severe cardiac and/or digestive. Previous results have shown that the immune response plays an important role, although no all mechanisms are understood. Immunoregulatory mechanisms such as apoptosis are important for the control of Chagas disease, possibly affecting the morbidity in chronic clinical forms. Apoptosis has been suggested to be an important mechanism of cellular response during *T. cruzi* infection. We aimed to further understand the putative role of apoptosis in Chagas disease and its relation to the clinical forms of the disease.

**Methods:**

Apoptosis of lymphocytes, under antigenic stimuli (soluble *T. cruzi* antigens – TcAg) where compared to that of non-stimulated cells. Apoptosis was evaluated using the expression of annexin and caspase 3^+^ by T cells and the percentage of cells positive evaluated by flow cytometry. In addition activation and T cell markers were used for the identification of TCD4^+^ and TCD8^+^ subpopulations. The presence of intracellular and plasma cytokines were also evaluated. Analysis of the activation status of the peripheral blood cells showed that patients with Chagas disease presented higher levels of activation determined by the expression of activation markers, after TcAg stimulation. PCR array were used to evaluate the contribution of this mechanism in specific cell populations from patients with different clinical forms of human Chagas disease.

**Results:**

Our results showed a reduced proliferative response associated a high expression of T CD4^+^CD62L^−^ cells in CARD patients when compared with IND group and NI individuals. We also observed that both groups of patients presented a significant increase of CD4^+^ and CD8^+^ T cell subsets in undergoing apoptosis after *in vitro* stimulation with *T. cruzi* antigens. In CARD patients, both CD4^+^ and CD8^+^ T cells expressing TNF-α were highly susceptible to undergo apoptosis after *in vitro* stimulation. Interestingly, the *in vitro* TcAg stimulation increased considerably the expression of cell death TNF/TNFR superfamily and Caspase family receptors genes in CARD patients.

**Conclusions:**

Taken together, our results suggest that apoptosis may be an important mechanism for the control of morbidity in *T. cruzi* infection by modulating the expression of apoptosis genes, the cytokine environment and/or killing of effector cells.

**Electronic supplementary material:**

The online version of this article (doi:10.1186/s12879-016-1523-1) contains supplementary material, which is available to authorized users.

## Background

Chagas disease, a neglected disease caused by *Trypanosoma cruzi*, remains a serious public health problem and affects about 10 million people in Latin America [1]. Chronic cardiomyopathy represents the most important and severe manifestation of human Chagas disease, eventually affecting approximately 20–30 % of individuals. The majority of the chronically affected individuals present the indeterminate (IND) form of the disease, with an apparent absence of morbidity [2, 3]. Epidemiological studies in endemic areas have shown that 2–5 % of patients will evolve each year from the indeterminate to the cardiac clinical form of the disease [4].

Although the pathophysiology of Chagas disease is not completely understood, it is widely accepted that the involvement of the immune response is critical in determining the disease outcome [5–8]. While the balance between inflammatory and anti-inflammatory cytokines produced by circulating cells in patients with IND form leans towards to an anti-inflammatory profile, patients with chagasic cardiomyopathy seem to display a predominantly inflammatory pattern [6, 9, 10]. The type of immune response induced in these individuals seems to be critical for the maintenance of a “healthy” balance between the parasite and the host [11]. In fact, several studies have demonstrated that immunoregulatory mechanisms are important for the control of infection, possibly affecting disease morbidity in chronic clinical forms [11, 12] such as T-cell suppression, polyclonal lymphocyte activation, and regulatory cytokines [13, 14]. Infection with *T. cruzi* leads to polyclonal lymphocyte activation [15], which, by itself, promotes T-cell apoptosis [16, 17]. In addition, antigens released by *T. cruzi,* such as *trans*-sialidase and HSP70, induce lymphocyte apoptosis [18, 19]. Therefore, it is possible that the parasite exploits host cell apoptosis to evade the immune response. Evidences also indicate that apoptosis plays a role in the resolution of inflammation [20].

In the present work, we evaluated the contribution of apoptosis in specific T cell populations on the development/maintenance of different clinical manifestations of human Chagas disease. Our findings demonstrated that *in vitro* stimulation with *T. cruzi* antigens induce lymphocyte apoptosis by continued cell activation, modulation of the expression of apoptosis genes and cytokine secretion profile. These findings may contribute to the regulation of immune response during human Chagas disease.

## Methods

### Study population

The patients that agreed to participate in this study were identified and selected from those being attended at the Referral Outpatient Center for Chagas Disease, which is located at the Clinics Hospital of the Federal University of Minas Gerais (UFMG), Brazil, under the medical care of one of us (MOCR). These patients were enrolled in a prospective cohort study initiated 20 years ago as previously described [13]. Patients infected with *T. cruzi* were grouped as indeterminate (IND) or with cardiomyopathy (CARD). The IND group included 15 asymptomatic individuals with age ranging from 24 to 66 years (mean of 39.6 ± 10.3), with no significant alterations in electrocardiography, chest X-ray and echocardiogram. The CARD group included 15 patients with age ranging from 23 to 69 years (mean of 48 ± 12.52) presenting dilated cardiomyopathy, characterized by the echocardiographic finding of a dilated left ventricle with impaired ventricular systolic function. Left ventricular ejection fraction (LVEF) and left ventricular diastolic diameter (LVDD) were used as clinical parameters of the ventricular function for Chagas disease patients, where LVEF <55 % and LVDD/body surface area ≥31 mm were used to define Chagas disease dilated cardiomyopathy [3]. None of the patients had undergone chemotherapeutic treatment, nor been previously treated for *T. cruzi* infection. Healthy individuals with age ranging from 29 to 55 years [mean of 42.6 ± 8.8), from a non-endemic area for Chagas disease with negative serological tests for the infection were included in the control group (non-infected NI).

### Ethics statement

This study was carried out in full accordance with all international and Brazilian accepted guidelines and was approved by the Ethics Committee of the René Rachou Research Center – FIOCRUZ (14/2006 CEPSH-IRR) and UFMG protocol COEP-ETHIC 001/79). All enrolled patients gave written informed consent prior to the inclusion in the study.

### *Trypanosoma cruzi* soluble antigen preparations

The CL strain of *T. cruzi* was used for antigenic preparation as described elsewhere [21]. After preparation, the protein concentration was determined, aliquoted and stored at - 70 °C prior use.

### Short-term *in vitro* whole blood cultures with *T. cruzi* antigens

Whole blood samples (final concentration of 1 × 10^6^ cells/mL) were treated with staurosporine (Sigma, St. Louis, MO, USA) (4 μM), soluble *T. cruzi* antigens (TcAg) (25 μg/mL) or untreated (stimulated with medium alone – RPMI 1640 supplemented with 1.6 % L-glutamine, 3 % antibiotic-antimycotic, 5 % of AB Rh-positive heat-inactivated normal human serum), and incubated for approximately 24 h at 37 °C in 5 % CO_2_. Following incubation, the cultures were treated with 220 μL of EDTA at 20 mM and maintained at room temperature for 15 min prior immunophenotypic staining for apoptosis assay, cell surface markers, and intracellular cytokine analysis.

### Cell preparation and proliferation assay

PBMC from Chagas patients and healthy individuals were isolated by Ficolldiatriazoate density gradient centrifugation (LSM; Organon Teknica, Charlesnton, S.C.) as previously described (Gomes, 2003). The cells were washed in RPMI 1640 medium and cultured in flat-bottom 96-well plates (Nunc Brand Products). Proliferative responses were evaluated by incubating 2.5 ×10^5^cells/well for TcAg (25 μg/mL) or 1.5 ×10^5^cell/well for mitogen stimulation (PHA, 10 μg/mL), respectively, in a final volume of 200 μL of complete RPMI-1640. Incubation was carried out in a humidified 5 % CO2 incubator at 37 °C for 3 days for PHA-stimulated cultures and 6 days for antigen-stimulated cultures. Cells were pulsed for the last 6 h of incubation with 1 μCi of [^3^H] methyl thymidine (PerkinElmer LAS, Shelton, CT, USA), and harvested onto glass fiber filters (Printed Filtermat A, Wallac, Finland). Radioactive incorporation was determined by liquid scintillation spectrometry (MicroBeta JET, PerkinElmer Inc., USA).

### Analysis of apoptosis profile

Short-term *in vitro* whole blood cultures were washed with 6 mL of FACS buffer (PBS supplemented with 0.5 % bovine serum albumin-BSA and 0.1 % sodium azide) by centrifugation at 600x*g* for 7 min at room temperature and resuspended in 5 mL of FACS buffer.

For the annexin V analysis, aliquots of 150 μL were transferred to polystyrene tubes and incubated for 30 min at room temperature (RT) with 2 μL of allophycocyanin (APC) – labeled anti-CD4 (RPA-T4) (BD Pharmingen) or anti-CD8 (RPA-T8) (BD Pharmingen) monoclonal antibodies. Following incubation, the red blood cells were lysed by the addition of 3 mL of FACS lysing solution (Becton Dickinson, CA, USA) for 10 min, and cells washed with 2 mL of PBS by centrifugation at 600 x*g* for 7 min at room temperature. The cells were resuspended in annexin V binding buffer (0.1 M Hepes/NaOH (pH 7.4) 1.4 M NaCl, 25 mM CaCl_2_ - Biosciences, San Jose, CA), for working solution (1X), then incubated for 15 min at RT (25 °C) in the dark with 5 μL annexin V-PE and 5 μL 7AAD. The reaction was stopped by the addition of 100 μL of 1x binding buffer for each tube.

For caspase-3 analysis, aliquots of 150 μL of whole blood cultures were transferred to polystyrene tubes and incubated for 30 min at room temperature with 2 μL of fluorescein isothiocyanate (FITC)-labeled anti-CD45 (2D1) (BD Biosciences) and 2 μL of peridinin chlorophyll-a protein (PerCP)-labeled anti-CD14 (M5E2) (BD Biosciences) monoclonal antibodies. The tubes were incubated in the dark for 10 min at RT. The cells were permeabilized in saponin buffer (0.5 %) (Sigma) for 15 min at RT in the dark. Finally, the cells were incubated with PE-conjugated rabbit anti-active caspase-3 mAb (C92-605) (BD Pharmingem) using 20 μL/1×10^6^ cells for 60 min at RT in the dark. Phenotypic analyses were performed by flow cytometry using a Becton Dickinson FACScalibur flow cytometer Analysis was performed on 7 × 10^4^ lymphocytes (gated according to their forward and side scatter properties. The sample acquisition and data analysis were performed using CellQuest software (BD Biosciences, USA).

### Analysis of cell surface markers and intracytoplasmic cytokines

Cultured cells were washed twice in PBS containing 1 % BSA and stained with monoclonal antibodies specific for cell-surface markers. Antibodies to CD4 (RPA-T4), CD8 (RPA-T8) and CD62L (DREG-56) (all from BD Pharmingen) were used. The cells were then fixed in formaldehyde (4 %) and permeabilized in saponin buffer (0.5 %) (Sigma, USA) for 15 min. Finally, the cells were incubated with anti-TNF-α (PE) (L293) (BD Biosciences) washed and ressuspended in FACS buffer prior acquisition in flow cytometer.

Phenotypic analyses were performed by flow cytometry using a Becton Dickinson FACScalibur flow cytometer, collecting data on 7 × 10^4^ lymphocytes gated according to their forward and side scatter properties. The sample acquisition and data analysis were performed using CellQuest software (BD Biosciences, USA).

### Detection of plasmatic cytokine levels by Cytometric Bead Array (CBA)

A cytometric beads array (CBA) immunoassay kit (BD Biosciences, USA) was used to measure cytokine levels (IFN-γ, TNF-α, IL-2 and IL-10) in plasma as described in previous studies [6]. The data were acquired in a Becton Dickinson FACScalibur flow cytometer and analyzed using BD CBA software (BD Biosciences, USA). The results were expressed by mean intensity of fluorescence (MIF).

### Apoptotic pathways triggered by *T. cruzi* infection in different clinical forms of Chagas disease

In order to determine putative apoptotic pathways triggered by *T. cruzi* infection, PBMC from infected patients IND (*n* = 2) and CARD (*n* = 2) were incubated only with culture medium- culture non-stimulated; or in the presence of antigen- culture stimulated with TcAg at a final concentration of 25 μg/mL. After 18 h of incubation at 37 °C and 5 % CO_2_ air, cells were recovered and washed with PBS. Subsequently, cells were submitted to a total RNA extraction protocol using NucleoSpin® RNA II kit (Macherey-Nagel, Germany). The total RNA was quantified according to standard procedures using spectrophotometer (Thermo Scientific, USA) and evaluated in agarose gel to confirm its integrity [22]. The cDNAs were obtained with Superscript II kit (Invitrogen, USA) using 120 ng of total RNA, according to manufacturer’s instructions. Afterwards, a RT-PCR reaction was performed using previously established constitutive human primers to confirm the cDNA synthesis.

The apoptotic transcripts were evaluated using a Human Apoptosis RT^2^ Profiler™ PCR Array kit (SABiosciences, USA) in a qPCR machine (7500, Applied Biosystem, USA), according to manufacturer’s instructions. Thirty one transcripts involved with pro-apoptotic activity were evaluated. Out of thirty one genes, twenty belong to TNF and TNF receptor superfamily, cell death domains and inductors of apoptosis; and eleven to the caspases family, which are also involved with pro-apoptotic activity. The qPCR data were analyzed by PCR Array Data Analysis Web Portal (SABiosciences, USA), and the results were expressed using the method of 2^-ΔΔC^.

### Statistical analyses

Statistical analyses were conducted using the R 2.15.0 software. Initially, the Anderson-Darling test was applied to verify whether the obtained data represent a normal distribution. Statistical comparative analyses were performed using the non-parametric: Mann–Whitney test to compare two groups (NI x IND or NI x CARD or IND x CARD); Kruskal-Wallis test to compare three groups (NI x IND x CARD) and, together with the Bonferroni correction (significance level, 0.05/3 = 0.0167). All tests were performed considering a significance level of 5 % (α = 0.05).

## Results

### Cellular proliferative response is decreased in CARD patients

**Fig. 1:**
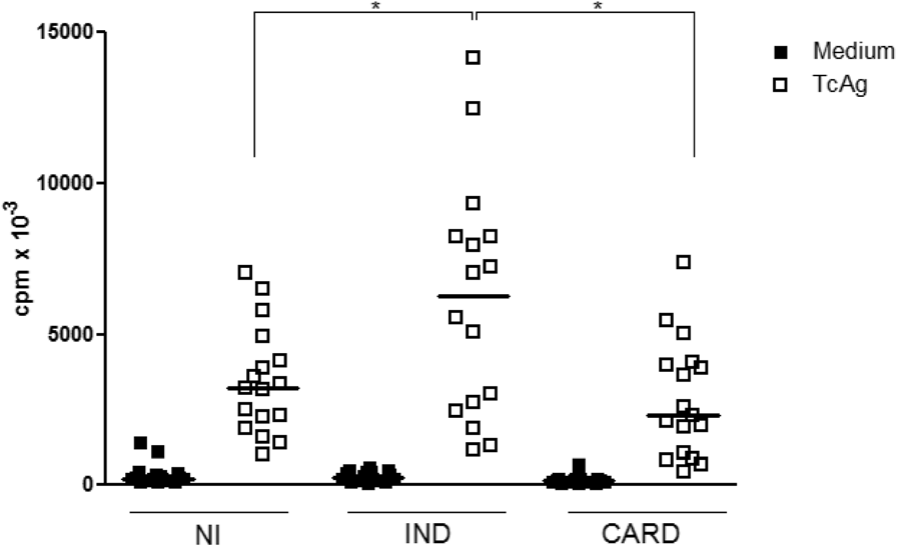
Proliferation of peripheral blood mononuclear cell induced by *T. cruzi* antigens (TcAg). A total of 3×10^5^ cells/well isolated from non-infected (NI) (*n* = 15), indeterminated (IND) (*n* = 15) and cardiac (CARD) (*n* = 15) groups were cultured in the presence of medium (RPMI-10 % AB serum) and TcAg (25 μg/mL) for 5 days, the last 18 h in the presence of 0.5 μCi[^3^H]-thymidine. The thymidine incorporation was measured by liquid scintillation spectroscopy. Data were reported as means of triplicates of counts per minute (cpm). Significant differences (*p*-value < 0.05) in the charts are identified by connecting lines and the symbol (*) for comparisons between the groups. Mann–Whitney test was used for comparison and the results were expressed as the median

The proliferative immune response was analyzed in PBMC from IND and CARD patients as well as NI individuals stimulated with soluble *T. cruzi* antigens (TcAg) by the quantification of H^3^-thymidine incorporation. We observed that PBMC proliferative response of the IND group had a significantly higher (*p* < 0.05) level of cellular proliferation after stimulation with TcAg when compared with CARD and NI groups (Fig. 1). No significant difference was observed in the proliferative response induced by PHA (data not shown).

### Increased downregulation of CD62L in CD4^+^ T cells is related to CARD patients

**Fig. 2:**
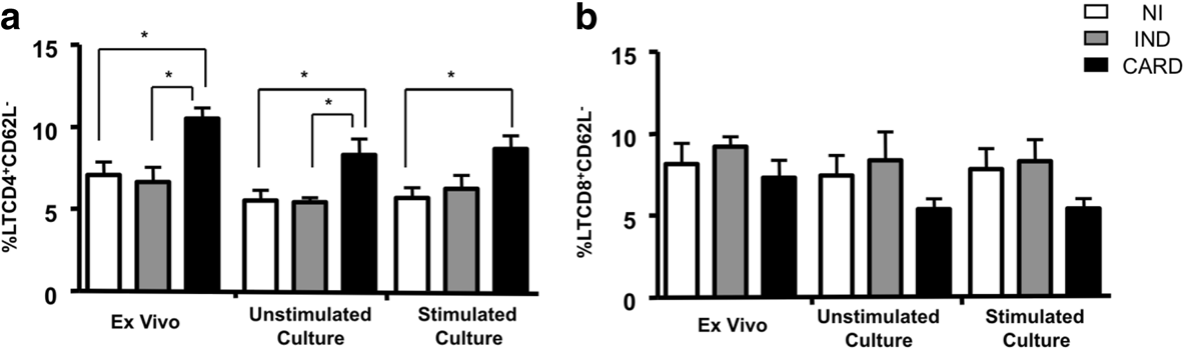
Analysis of percentage of downregulation of CD62L by CD4^+^ (**a**) and CD8^+^ (**b**) T cell subsets in the peripheral blood from chagasic patients, in *ex vivo* context and after *in vitro* stimulation with *T. cruzi* antigens, from patients with distinct clinical forms of Chagas disease: indeterminate – IND (*n* = 9, gray bars); cardiac – CARD (*n* = 14, black bars) and non-infected individuals – NI (*n* = 15, white bars). The results were expressed as mean ± standard deviation (SD). Significant differences (*p* < 0.05) in the charts are identified by connecting lines for comparisons between groups

We also evaluated the loss of expression (CD62L^−^) in circulating CD4^+^ and CD8^+^ T cells, before and after *in vitro* stimulation with TcAg. The analysis showed significant increase on the percentage of CD4^+^CD62L^−^ cells in CARD patients (*p* < 0.05) in *ex vivo* analysis as well as non-stimulated cultures when compared with NI and IND groups (Fig. 2a). Moreover, after TcAg *in vitro* stimulation, a higher percentage of CD4^+^CD62L^−^ cells in CARD patients (*p* < 0.05) it was also observed when compared to NI individuals (Fig. 2a). *Ex vivo* expression of CD62L^−^ by CD8^+^ cells and after in vitro culture in the presence or not of TcAg did not show any statistically significant differences between the study groups (Fig. 2b).

### Lymphocytes from IND and CARD patient presented elevated frequency of apoptosis after *in vitro* TcAg stimulation

The low proliferative response and high percentage of CD4^+^CD62L^−^ T cells observed in CARD patients might be related to regulatory mechanisms involved in the control of the immune response induced by *T. cruzi*. In this context, apoptosis has been previously demonstrated as play a role in experimental model of acute *T. cruzi* infection [23–26] and also in human heart tissues [27]. In order to investigate if apoptosis might be a factor related to the lower proliferative response and if it is caused by high T cell activation in the CARD patients, the frequency of annexin V^+^ and active caspase 3^+^ was analyzed as markers of apoptosis.

**Fig. 3:**
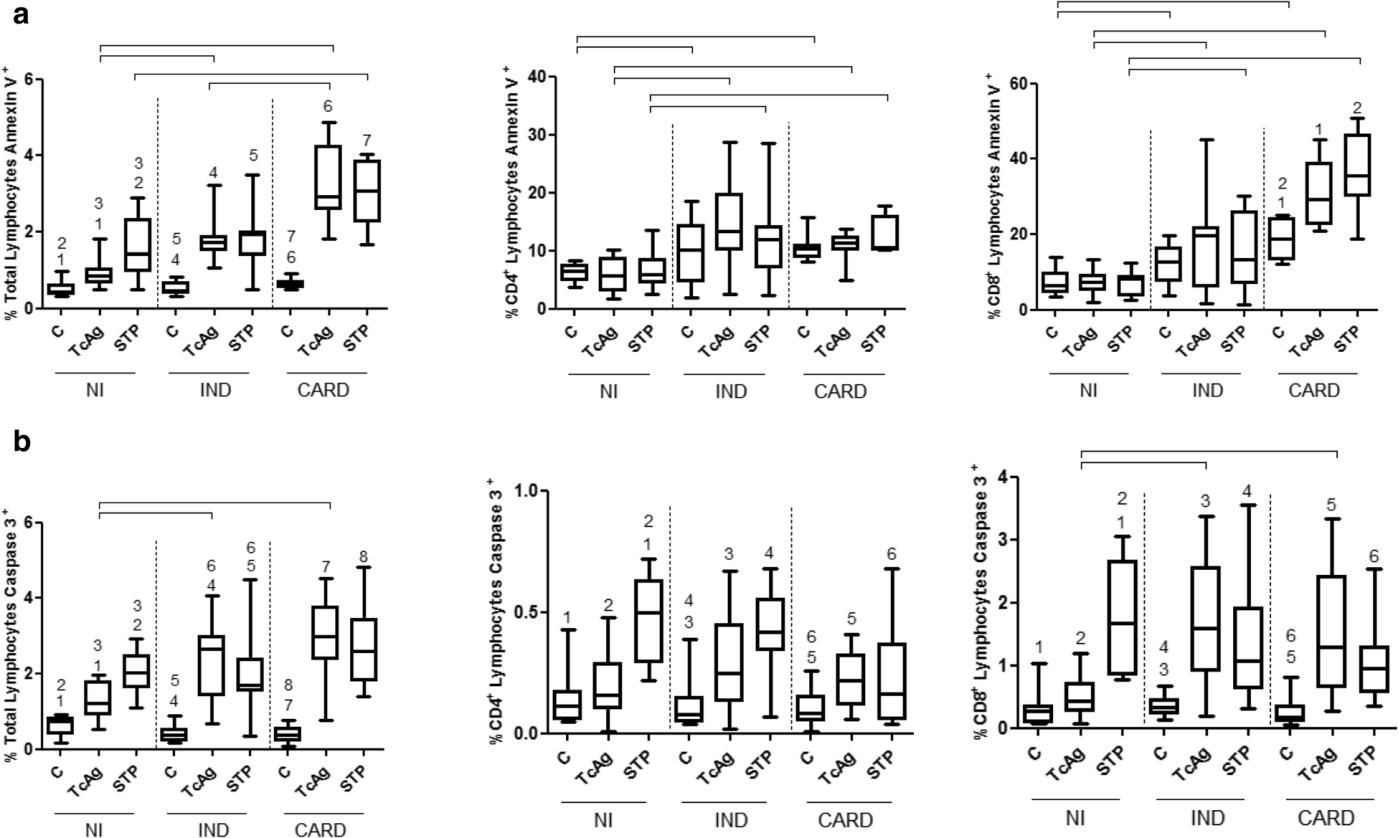
The apoptotic profile of T lymphocytes from peripheral blood of uninfected individuals (NI) and patients with Chagas disease - indeterminate (IND) or cardiac (CARD) form. The detection of apoptosis was performed using the percentage of annexin^+^ (panel **a**) and caspase 3^+^ (panel **b**) in total lymphocytes, CD4^+^ or CD8^+^ T cell subsets. The lymphocytes were evaluated without stimulation - control cultures (C) and after *in vitro* stimulation with TcAg (*T. cruzi* antigens) and STP (staurosporin). Mann–Whitney test was used for comparison and the results were expressed as the median with interquartile range. Differences (*p* < 0.05) are presented by the corresponding numbers and connecting lines in the box plot graphs

Our data show that after *in vitro* stimulation with TcAg the lymphocytes from CARD displayed high frequency of annexin V^+^ when compared with IND and NI groups (Fig. 3a). The higher frequency of annexin V^+^ in CD4^+^ T cells was observed in IND and CARD patients as compared to NI (Fig. 3b). On the other hand, lower frequency of annexin^+^ in CD8^+^ T cells were observed in IND and NI groups as compared with CARD group (Fig. 3c). A similar profile was observed for the cultures in the presence of Staurosporin-STP, a positive control for apoptosis.

The frequency of total lymphocytes and CD4^+^ or CD8^+^ T cells subsets from IND and CARD patients exhibited similar apoptosis profile as compared with NI individuals when active caspase 3^+^ was analyzed (Fig. 3a and b, respectively).

### Activated Lymphocytes^+^ TNF-α^+^ form CARD patients were susceptible to apoptosis

**Fig. 4:**
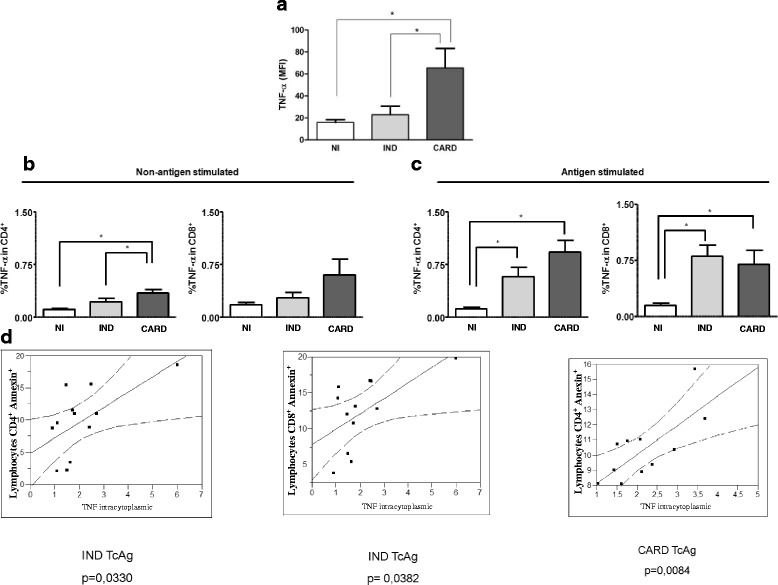
Analyses of cytokine levels and their association with cardiac morbidity expressed by the clinical classification. (**a**) The analysis of plasma cytokine levels was performed as described in material and methods. The groups evaluated were: NI (*n* = 15, white box), IND (*n* = 15, light gray box), and CARD (*n* = 15, dark gray box). The results were expressed by mean intensity of fluorescence (MIF). Plasma TNF-α levels in NI, IND, and CARD groups and their association with cardiac morbidity. (**b**, **c**) Analysis of percentage of CD4^+^TNFα^+^ and CD4^+^TNFα^+^ T cells in the peripheral blood from chagasic patients, in *ex vivo* context and after *in vitro* stimulation with *T. cruzi* antigens, from patients with distinct clinical forms of Chagas disease. Significant differences (*P*-value < 0.05) in the charts are identified by connecting lines and the symbol (*) for comparisons between the groups. (**d**) Correlation analysis between percentage of annexin^+^ CD4^+^ and annexin^+^ CD8^+^ T cells and TNF-α intracytoplasmic in the IND (*n* = 15) and CARD (*n* = 15) groups. Mann–Whitney test was used for comparison and the results were expressed as the median. Correlation analysis were done using the Spearman correlation coefficient, and the results were considered significant with a *p*-value < 0.05. Significant differences (*P*-value) are indicated in each graph together with the *r* values

A second hypothesis to explain the induction of programmed cell death in lymphocytes from chagasic patients involves the cytokine environment. It is known that TNF-α is a major cytokine implicated in the apoptosis pathway. Thus, we have further evaluated serum levels and intracytoplasmic expression of this cytokine *ex vivo* and following incubation with TcAg. We observed that plasma from CARD patients presented a higher expression of TNF-α than IND and NI groups (*p* < 0.05). Also, after TcAg stimulation, chagasic patients (IND and CARD) presented a higher frequency of TNF-α by total lymphocytes (Fig. 4b), and CD4^+^ T or CD8^+^ T cells when compared with NI groups (Fig. 4c and d, respectively). Analysis of serum levels of IFN-γ and IL-10 were also performed. IFN-γ was elevated in sera from cardiac patients and IL-10 in indeterminate patients as previously shown [28]. In this study we observed that IL-2 was also elevated in sera from cardiac patients (Additional file 1). These results did not show any association with apoptosis.

To determine whether TNF-α expression by total lymphocytes and CD4^+^ or CD8^+^ T cells subsets is associated with the frequency of apoptosis, we performed a correlative analysis between the frequency of apoptosis and TNF-α expression. We observed a positive and significant correlation between high frequency of annexin^+^ in CD4^+^ T cells and high frequency of TNF-α in CD4^+^T cells both in IND and CARD groups. Moreover, a significant positive correlation was found, between frequency of annexin^+^CD8^+^ T cells and TNF-α^+^CD8^+^T cells in CARD group (Fig. 4d). Together, these results suggested that both CD4^+^ and CD8^+^ T cells from CARD patients expressing TNF-α were highly susceptible to undergo apoptosis upon *in vitro* stimulation.

### Cell apoptosis in CARD patients might be induced by TNF receptors superfamily and/or caspase family pathway

Once we showed that CARD patients presented higher frequency of apoptotic cells, the next step was to identify the possible apoptotic pathway used to activate T apoptosis in T cells from patients with this clinical form, before and after *in vitro* stimulation with TcAg antigen. Initially, 31 apoptosis-related genes from TNF/TNFR superfamily and Caspase family were grouped and categorized according to their respective families and function. Analysis of the others 53 genes of human apoptosis pathway were also performed and these results did not show any significant differences in all groups (data not shown).

**Fig. 5:**
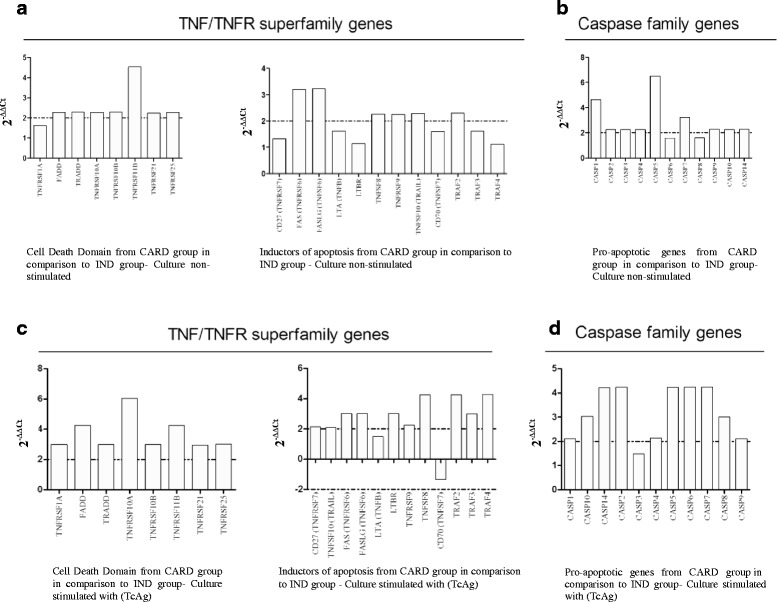
Apoptotic pathways presented by PBMCs from *T. cruzi*-infected patients, displaying different clinical forms of the Chagas disease. Relative analysis of genes expression from TNF/TNFR superfamily members, and Caspase family members were evaluated. The fold-change values (2^-ΔΔCt^) were represented by the bars, which show the expression of apoptotic genes from *T. cruzi*-infected patients presenting the cardiac clinical form (CARD) in comparison to *T. cruzi*-infected patients presenting the indeterminate clinical form (IND). **a** and **b**- Culture non-stimulated; **c** and **d**- Culture stimulated with TcAg

In the absence of *in vitro* TcAg stimulation, when we evaluated the TNF/TNFR superfamily genes from CARD group was determined. The results showed that cell death receptors (FADD, TRADD, TNFRSF10A (TNF receptor superfamily member 10A), TNFRSF10B, TNFRSF11B, TNFRSF21 and TNFRSF25) were up-regulated when compared with IND group (Fig. 5a). Moreover, other pro-apoptotic genes (FAS, FASL, TNFSF8, TNFRSF9, TRAIL, TRAF2) were also up-regulated in CARD patients when compared with individuals from the IND group (Fig. 5a). When also evaluated the caspase family genes, the results showed that caspase 1 (CASP1), caspase 2 (CASP2), caspase 3 (CASP3), caspase 4 (CASP4), caspase 5 (CASP5), caspase 7 (CASP7), caspase 9 (CASP9), caspase 10 (CASP10) and caspase 14 (CASP14) genes, were up-regulated in CARD group in comparison with IND patients (Fig. 5b).

Interestingly, the *in vitro* TcAg stimulation increased considerably the expression of cell death TNF/TNFR superfamily as well as caspase family receptors genes (FADD, TRADD, TNFRSF10A, TNFRSF10B, TNFRSF11B, TNFRSF21, TNFRSF25, TNFSF10, FAS, FASLG, TNFRSF9, TNFSF8, TRAF2, TRAF4) (Fig. 5c and d, respectively). Furthermore, high expression of genes such as TNFRSF1A, CD27, LTA, TRF3, TRF4 and CASP3 were observed after PBMCs cultures from CARD when compared to IND patients (Fig. 5c and d, respectively). Moreover, the CD70 gene was downregulated in CARD patients when compared to IND patients (Fig. 5c).

## Discussion

In the present work, we have shown that apoptosis is associated with reduced proliferative response, a high expression of T CD4^+^CD62L^−^ cells, an increase TNF-α intracellular production and expression of genes of cell death TNF/TNFR superfamily and caspase family in CARD patients.

In this context, some studies have been shown that CARD patients presented low proliferation of T cells when compared with healthy and non-chagasic cardiomyopathy donors [29–32]. Although, the mechanisms of proliferative dysfunction in Chagas disease need further investigation, studies suggest that they could be related to the decrease of co-stimulatory molecules expression, receptor cytokine starvation or expression of inhibitory receptors as PD-1 [12, 32, 33].

Some studies suggest that activated cells are susceptible to apoptosis, which may represent a mechanism of immunoregulation [34, 35]. To assess the activation status of T cell subsets, we evaluated whether CD62L is downregulated in these cell subpopulations. Our data showed that CARD patients presented a significantly higher percentage of TCD4^+^CD62L^−^ cells suggesting that a putative involvement of this cell type in the exacerbation of the immune response to the parasite and, consequently, on the development of myocarditis by cell death induced by activation. Dos Santos et al*.* [36] showed that the majority of TCD4 and TCD8 lymphocytes in the inflammatory foci from the heart of chagasic patients did not express or slightly expressed CD62L and T CD8^+^ cells are the majority of the activated cells in the tissue when compared with CD4^+^ T cells. The inflammatory process occurring in Chagas’ disease mainly consists of CD8 T lymphocytes, CD4 T lymphocytes and macrophages. The extent of inflammatory reaction and the tissue damage caused may contribute to loss of myocardial cells, and to the heart failure that is observed on more severe cases of chronic Chagas’ disease. Tostes Jr et al*.* [37] showed that myocardial cell loss by apoptosis and fibrosis contributes to heart failure in the chronic phase of Chagas’ disease. In fact, apoptosis has been considered a cause of heart failure in other diseases such as myocardial infarction and heart hypertrophy [38, 39], as well as a mechanism involved in the control of the immune response in experimental models [26]. On the other hand, heart failure by itself may induce apoptosis [40].

In this work, high expression of annexin by lymphocytes from CARD group was observed when compared with IND and NI groups, as well as an increase on the expression of annexin by CD4^+^ T cells and caspase by both CD4^+^ and CD8^+^ T cells by chagasic patients when compared with NI group after *in vitro* stimulation with *T. cruzi* antigens. In Chagas’ disease, the occurrence of apoptosis in T lymphocytes was observed after antigen stimulation in experimental models [26] although its significance in terms of clinical form or outcome of the disease was not clear. Apoptosis-like death has been reported in amastigote nests and trypomastigotes forms from *T. cruzi*, and this mechanism has been associated with control of parasite burden regulated by the parasite itself or by the host, parasite evasion of the host’s immune response and clonal selection [41–44]. Together, these findings suggest that although the lymphocytes from CARD patients presented lower proliferative response upon antigenic recall, lymphocytes from IND and CARD patients presented a singular ability to undergo apoptosis that may reflect different regulatory mechanism.

Rodrigues et al. [45] demonstrated a high percentage of lymphocyte apoptosis in patients with a severe cardiomyopathy, associating this event to activation of programmed death pathways, by Fas/Fas-L or TNF-α receptors, leading to parasite escape, and consequently, to a continuous stimulation of the immune system. In this context, T cells apoptosis in experimental model leads to an increase of parasite growth [46]. Indeed, apoptosis has been suggested to be an important mechanism to control the immune response and heart damages [47].

In order to determine whether TNF-α contributes to T cell apoptosis, the plasmatic and intracellular production by T cell subsets was evaluated. Our data demonstrated that CARD group showed increased levels of circulating and intracytoplasmic TNF-α, and up-regulation of the TNF receptor gene superfamily. Lula et al. [48] have shown correlation among soluble ligands of TNF superfamily (TNF-α, TRAIL and FasL/CD95L) and functional disorders of the left ventricle in chronic chagasic patients with cardiomyopathy. These results are associated with ligand receptors associated with programmed cell death, suggesting that apoptotic mechanisms are involved with the development of miocardiopathy in Chagas disease.

Ferreira et al. [49] showed a correlation between high serum levels of TNF-α and the occurrence of severe Chagas cardiomyopathy. Also, it has been shown that there is an inverted correlation between high levels of TNF-α with the lowest left ventricular ejection fraction seen in patients with chronic Chagas cardiomyopathy [50]. Moreover, patients with heart failure are shown to have a significantly lower PBMC proliferative response but higher levels of apoptosis and Fas and Fas-L expression [45]. TNF-α may play a role on the high levels apoptosis and in the low proliferative response observed in patients with heart failure. TNF-α may contribute to the induction of apoptosis by the interaction with its receptor or by induction of Fas and Fas-L expression [51]. Our results suggest that the high levels of TNF-α detected in plasma from CARD group may contribute to the heart condition. Additionally, our data support the hypothesis that high levels of TNF-α and up-regulation of the TNF receptor gene superfamily lead to an increase in apoptosis, and consequently the exacerbation of the pathology.

## Conclusions

Here, we showed that apoptosis is associated with low proliferative response, intense T cell activation, high TNF-α production and up-regulation of genes associated with TNF receptors superfamily and caspase family in CARD ’patients. These results suggest that apoptosis could interfere on the development and/or maintenance of the different clinical forms of Chagas disease. Assuming that the immunological regulation in the IND group, may control the development of Chagas cardiomyopathy, the absence of this mechanism in the CARD group, may be one of the factors associated with sustained inflammation which would, consequently, lead to a higher morbidity in the latter group. The association of lymphocyte apoptosis, induced by the constant activation of the immunological system, with high levels of inflammatory cytokines and associated pathological events (fibrosis and apoptosis) may contribute with the development and progression of heart injuries in the chronic phase of the human Chagas disease.

## Additional file

Additional file 1: Figure S1. Analyses of plasma cytokine levels expressed by the clinical classification. The analysis of plasma levels was performed as described in material and methods. The groups evaluated were: NI (*n* = 15, white box), IND (*n* = 15, light gray box), and CARD (*n* = 15, dark gray box). The results were expressed by mean intensity of fluorescence (MIF). (**A**) Plasma TNF-α levels in NI, IND, and CARD groups. (**B**) Plasma IL-2 levels in NI, IND, and CARD groups. (**C**) Plasma IFN-γ levels in NI, IND, and CARD groups. (**D**) Plasma IL-10 levels in NI, IND, and CARD groups. Significant differences (*P*-value < 0.05). Statistical comparative analyses were performed, in groups of two, between the NI, IND, and CARD groups, using the non-parametric Kruskal-Wallis test and Mann–Whitney U test, together with the Bonferroni correction (significance level, 0.05/3 = 0.0167). The letters represent statistically significant differences (*p < 0.05*) between the groups: a = difference when compared to NI group; b = difference when compared to IND group; c = difference when compared to CARD group. (PDF 113 kb)

## Abbreviations

CARD: cardiac clinical form of chagas disease
CASP: caspase family genes
DEATH Domain Proteins: FADD, TNFRSF10A, TNFRSF10B (DR5), TNFRSF11B, TNFRSF21, TNFRSF25 (DR3), TRADD
FACS: fluorescence-activated cell sorter
FasL: fas ligand
FITC: fluorescein isothiacynate
HSP70: 70 kilodalton heat shock proteins
IFN-γ: interferon-gamma
IL-10: interleukin 10
IL-2: interleukin 2
IND: indeterminate clinical form of chagas disease
LVDD: left ventricular diastolic diameter
LVEF: left ventricular ejection fraction
mAb: monoclonal antibodies
NI: non-infected
PBMC: peripheral blood mononuclear cells
PCR: polymerase chain reaction
PerCP: peridinin chlorophyll protein complex
PHA: Phytohaemagglutinin
RT: room temperature
RT- qPCR: Relative quantification in real-time-polymerase chain reaction
RT-PCR: reverse transcription-polymerase chain reaction
STP: Staurosporin
TcAg: soluble *T. cruzi* antigens
TCD4^+^: helper T cells
TCD8^+^: cytotoxic T cell
TNF: tumor necrosis factor
TNF/TNFR Domain Proteins: FAS (TNFRSF6), FASLG (TNFSF6), TNFSF10 (TRAIL), TNFRSF10A, TNFRSF10B (DR5), TNFRSF11B, TNFRSF21, TNFRSF25 (DR3), TNFRSF9
TNFR: tumor necrosis factor receptor
TNF-α: tumor necrosis factor alpha
TRAF domain proteins: TRAF2, TRAF4
UFMG: Universidade Federal de Minas Gerais
