# Supplementary figures and images for: Immunoregulatory mechanisms in Chagas disease: modulation of apoptosis in T-cell mediated immune responses
Source: BMC Infect Dis. 2016 Apr 30;16:191. doi: 10.1186/s12879-016-1523-1 (PMC4852404; doi:10.1186/s12879-016-1523-1)

Supplemental Figure 1

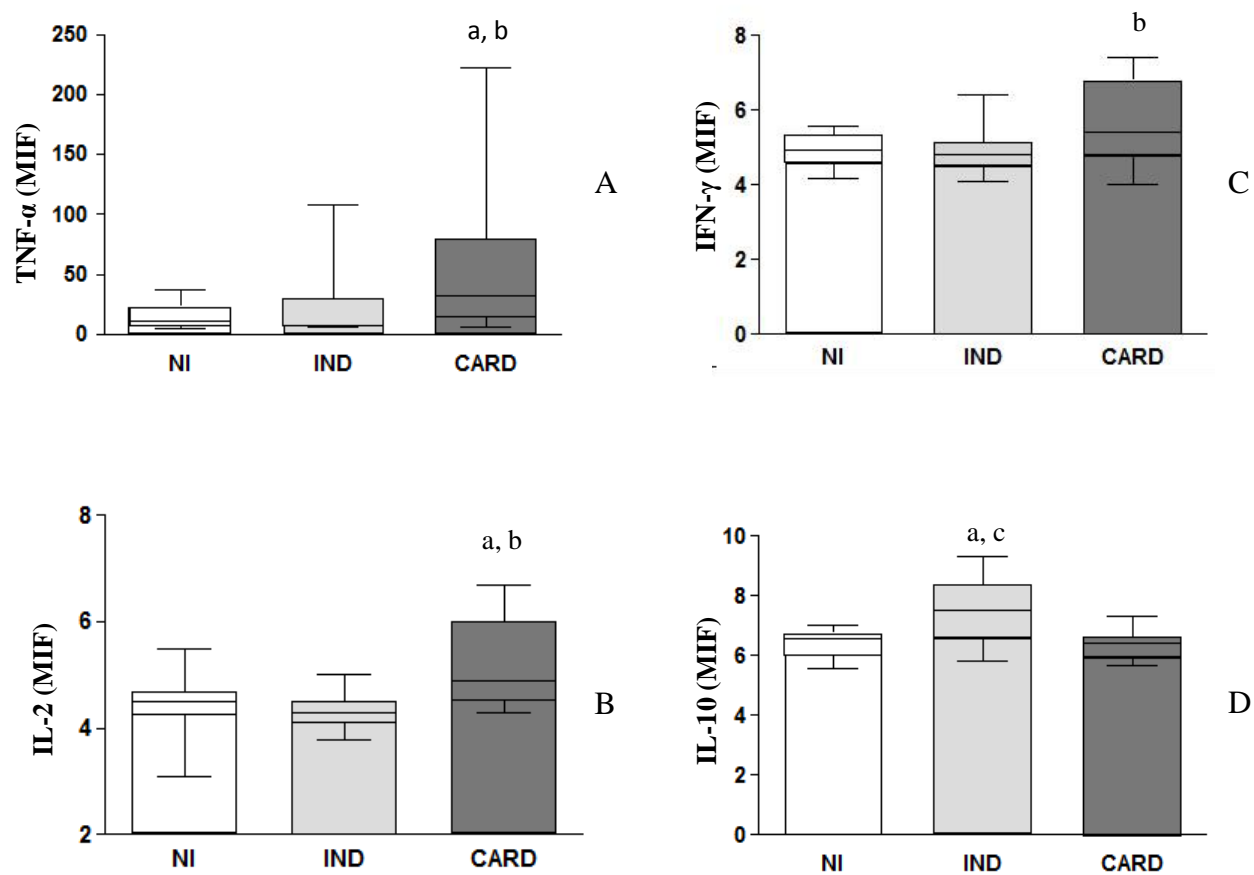

Supplement: Additional file 1: Figure S1. — Analyses of plasma cytokine levels expressed by the clinical classification. The analysis of plasma levels was performed as described in material and methods. The groups evaluated were: NI (n = 15, white box), IND (n = 15, light gray box), and CARD (n = 15, dark gray box). The results were expressed by mean intensity of fluorescence (MIF). (A) Plasma TNF-α levels in NI, IND, and CARD groups. (B) Plasma IL-2 levels in NI, IND, and CARD groups. (C) Plasma IFN-γ levels in NI, IND, and CARD groups. (D) Plasma IL-10 levels in NI, IND, and CARD groups. Significant differences (P-value < 0.05). Statistical comparative analyses were performed, in groups of two, between the NI, IND, and CARD groups, using the non-parametric Kruskal-Wallis test and Mann–Whitney U test, together with the Bonferroni correction (significance level, 0.05/3 = 0.0167). The letters represent statistically significant differences (p < 0.05) between the groups: a = difference when compared to NI group; b = difference when compared to IND group; c = difference when compared to CARD group. (PDF 113 kb) [file 12879_2016_1523_MOESM1_ESM.pdf]
